# Improvement of quality of life following 6 months of methadone maintenance therapy in Malaysia

**DOI:** 10.1186/1747-597X-7-32

**Published:** 2012-08-01

**Authors:** Nizam Baharom, Mohd Rohaizat Hassan, Norsiah Ali, Shamsul Azhar Shah

**Affiliations:** 1 Department of Community Health, Malaysian National University Medical Centre, Jalan Yaacob Latif, 56000 Cheras, Kuala Lumpur, Malaysia; 2 Tampin Health Clinic, District of Tampin, Negeri Sembilan, Malaysia

**Keywords:** Methadone, Quality of life, Malaysia

## Abstract

**Background:**

Methadone Maintenance Therapy (MMT) is one of the popular choices for drug substitution therapy and is fairly new in Malaysia. Aside from its role in harm reduction against HIV infection, MMT programme may potentially enhances clients’ quality of life. This study aims to identify the impact of MMT programme on clients’ quality of life after 6 months in treatment and to explore factors that may be associated with changes in their quality of life.

**Methods:**

In this retrospective report review, 122 subjects from 2 government MMT clinics were selected from the district of Tampin, Negeri Sembilan, Malaysia. The raw score from the WHO Quality of Life questionnaire (WHOQOL-BREF), at baseline and 6 months after therapy were collected and converted to 0–100 scale form to give quality of life scores for four domains; physical, psychological, social relationships and environment. Other variables of interest were socio-demography, age when joining MMT programme, age and duration of illicit drug use, HIV and Hepatitis C status, and the Opiate Treatment Index (OTI) score on drug use, sexual and social aspect at the baseline. Statistical analysis used the SPSS version 16.

**Results:**

There was significant improvement in all four domains of quality of life, after 6 months of MMT. The largest improvement was for psychological domain (mean score difference 15.54 ± 20.81). Multivariable linear regression analysis showed that, for the physical domain, there was no significant predictor. For both the psychological and social domains, having tertiary education is a significant predictor for improvement in both aspects of quality of life. Negative HIV status is associated with improvement for the environment domain.

**Conclusions:**

There was a significant short term improvement in the quality of life of MMT clients who stayed in the programme for at least 6 months in the district of Tampin, Negeri Sembilan, Malaysia.

## Background

Substance abuse is a global problem and a major public health concern for many nations. In developing countries, one of the reasons for the concern is the emerging of HIV epidemics among injecting drug users. Among the options for substitution therapy, methadone has been recognised as one of the most popular choices. In 2004, WHO had proposed for methadone as an essential medicine for the management of opioid dependence [1]. Methadone is a long acting synthetic opioid, previously used predominantly as pain reliever, but has found its role in opioid substitution therapy or specifically Methadone Maintenance Therapy (MMT) [2,3].

There is strong evidence that drug substitution therapy facilitates the prevention of HIV transmission as highlighted by Sorensen and Copeland [2]. In Hong Kong, the prevalence of HIV among opioid abusers remains low since the introduction of methadone as drug substitution therapy in 1980s [4]. Part of the reasons is due to reduction in high risk behaviours such as needle sharing. There is also evidence that stopping the misuse of injecting drugs slows the progression of HIV disease among infected persons [5]. The accomplishment of MMT programme can also be attributed to the non-pharmacological component of the treatment [3,6-8].

In Malaysia, substance abuse has been around since pre-independence era and in 1980s, heroin abuse was considered as a national threat [9,10]. Previous rehabilitation programmes for drug addicts were yielding little success, and because of that, MMT programme was introduced in 2005 as part of the harm reduction programme [9,11]. Higher level of compliance was found in MMT programme in comparison to previous rehabilitation therapies [9,12]. Even though MMT is relatively new in Malaysia, the positive outcome showed so far has encouraged the government to launch more harm reduction centres throughout the country [11].

The MMT clinics in the district of Tampin, Malaysia were some of the pioneer government MMT services. It started out with just one small clinic in 2006. Now, there are two clinics attending to about 150 clients. Both MMT clinics are managed by a Family Medicine Specialist. In 2008, Tampin Health Clinic received acknowledgement by the World Health Organisation for good management and implementation of its methadone replacement therapy programme.

The interest of measuring quality of life has increased in the recent decades as it is seen as a tool for impact measurement in any intervention [13,14]. Particularly so in managing chronic diseases such as drug dependency, where one of the aims of treatment is to enhance the quality of life by reducing the impact of the disease [15]. In Malaysia, routine quality of life assessment is part of the MMT programme as indicated in the Ministry of Health’s National Policy and Standard Operating Procedures for Methadone Maintenance Therapy [16]. This assessment utilizes the WHO Quality of Life questionnaire, the WHOQOL-BREF, which assess what the client feels about his/her life for the past two weeks. It is an abbreviated version of the WHOQOL-100, and contains 24 questions assessing four domains of quality of life; physical, psychological, social relationships and environment, as well as 2 additional questions on overall quality of life and general health [17,18]. The WHOQOL-BREF is available in 19 languages [18] and the Malay version is used in Malaysian MMT programme [16][19]. The Malay version of WHOQOL-BREF has been validated before in a hospital setting (with Cronbach alpha ranging from 0.64 in the psychological domain to 0.80 in physical domain) and showed high correlation with the WHOQOL-100 [19].

The objectives of this study were to identify the impact of MMT programme on clients’ quality of life after 6 months of treatment and to explore factors that may be associated with changes in quality of life for MMT clients. As the MMT programme is fairly new in Malaysia, evidence for its effectiveness is still very limited and indistinct.

## Methods

### Study design

The study design was a report review research. The subjects were from two MMT clinics; the Tampin Health Clinic and Gemas Health Clinic, in a district of Tampin, Negeri Sembilan, Malaysia.

The clients’ report files from the two MMT centres were reviewed. The study was conducted in April 2010. The inclusion criteria were that client must be registered with the MMT clinic and had completed the WHOQOL-BREF assessment at baseline and 6 months after enrolment in MMT programme. Subjects who had not completed WHOQOL-BREF assessment at baseline and 6 months and those who were terminated from the programme before 6 months of treatment, were excluded in this study.

The administration of WHOQOL-BREF at baseline to MMT clients was assisted by trained health personnel to ensure that clients fully understand the questionnaire. Subsequent assessments at 6, 12, 18 and 24 months were completed by the clients themselves, unless assistance was required for reasons such as illiteracy and not understanding written Malay. During the study period, 57 clients have completed methadone therapy for one year or more.

Social demographic data such as age at the time of enrolment, ethnicity, gender, marital status, employment and formal education were noted. Other variables of interest were the age of starting illicit drug, duration of illicit drug use, HIV status (ELISA for HIV-1 and HIV-2 antibodies), Hepatitis C status (anti-HCV), numbers of positive urine tests (for illicit drugs) in the first 6 months of MMT, and the Opiate Treatment Index (OTI) drug use, sexual and social score at baseline (during the initiation of treatment). OTI is a standardized set of measures for the evaluation of opiate treatment [20]. Higher score denotes a greater degree of dysfunction at that particular segment or domain. OTI assessment is done routinely at baseline and other preset intervals during MMT treatment. Ethical approval was given by the Ministry of Health, Malaysia, reference: KKM/NIHSEC 07/0703 02Jld16.

### Statistical analysis

The scoring from the WHOQOL-BREF (raw score) was transformed to the 0–100 scale scoring format, according to the guideline for the transformation of raw WHOQOL-BREF score, which is available from the WHOQOL Group [21]. The end result gave scores in the transformed 0–100 scale format for each of the four domains in quality of life; physical, psychological, social relationships and environment. The statistical analysis was done using the SPSS programme version 16.0. Dependent *T*-test was used to determine the significance of the difference between the quality of life scoring at 0 and 6 month. Pearson correlation and ANOVA test were performed to identify variables associated with such difference in quality of life.

## Results

### Sociodemography characteristics of subjects

All 122 subjects were male and predominantly Malay (92.6%). The age of subjects when joining the MMT programme ranged from 18 to 58 years old with mean of 38.4 ± 7.96. 78.7% of subjects have full time employment and most of them were working as rubber tappers (in agriculture). The socio-demography characteristics of subjects are listed in Table 1. 15 subjects were excluded from this study.

**Table 1:** Socio-demographic characteristics of the subjects (n = 122)

	**frequency**	**%**
Gender		
Male	122	100.0
Age when joining MMT (years)		
< 20	1	0.8
20 – 29	15	12.3
30 – 39	53	43.4
40 – 49	43	35.2
50 – 59	10	8.2
Overall mean = 38.4 ± 7.96		
Ethnicity		
Malay	113	92.6
Chinese	2	1.6
Indian	7	5.7
Marital status		
Single	52	42.6
Married	52	42.6
Divorced	17	13.9
Widower	1	0.8
Employment status		
Unemployed	11	9.0
Part time employment	15	12.3
Full time employment	96	78.7
Educational level		
Primary	9	7.4
Junior secondary	44	36.1
Senior secondary	65	53.3
Tertiary education	4	3.3
Age starting illicit drug use (years)		
11 – 15	19	15.6
16 – 20	66	54.1
21 – 25	32	26.2
> 25	5	4.1
Overall mean = 19.3 ± 3.65		
HIV status		
Negative	95	77.9
Positive	21	17.2
Unknown	6	4.9
Hepatitis C status		
Negative	22	18.0
Positive	89	73.0
Unknown	11	9.0

### Quality of life after 6 months of MMT

Paired *t*-test was done on the WHOQOL score at baseline and 6 months after, for all four domains (Table 2). The results showed significant positive mean differences in all four domains with p values ≤ 0.001. The largest mean difference was for psychological domain, with mean improvement of 15.54 ± 20.81. Social domain showed the least improvement in WHOQOL scores (7.32 ± 22.71).

**Table 2:** **Paired*t*-test of quality of life scores by domain at baseline and at 6 month**

**Domain**	**Baseline score**	**6 month score**	**Difference (mean ± SD)**	**t**	**p value**
	**(mean ± SD)**	**(mean ± SD)**			
Physical	51.54 ± 15.81	65.01 ± 11.83	13.47 ± 16.83	−8.840	< 0.001*
Psychological	50.18 ± 17.58	65.72 ± 13.33	15.54 ± 20.81	−8.250	< 0.001*
Social	53.09 ± 22.24	60.41 ± 19.02	7.32 ± 22.71	−3.559	0.001*
Environment	55.84 ± 12.47	65.39 ± 12.63	9.55 ± 15.30	−6.896	< 0.001*

*significant when p value < 0.05.

### Factors associated with changes in quality of life

For each domain of the quality of life, univariate analysis was done to identify variables that are associated with changes in the domain. Significant results for each domain are shown in Table 3. There is a significant (but poor) correlation between number of positive urine tests and score difference in the physical domain (r = 0.213, p = 0.019). ANOVA bivariate analysis showed significant difference between education levels and mean score differences in psychological and social relationship domains. Post hoc test (Scheffe) revealed that there was a significant difference between tertiary level against primary level education. The HIV negative clients showed significantly better improvement in psychological and environment domains (post hoc tests were significant between HIV negative and HIV positive means, in both domains). There were no significant correlation found between the OTI score with any of the quality of life domain.

**Table 3:** Univariate analysis showing only significant variables associated with improvement in each quality of life domain

**Variables (n)**	**Domain 1**		**Domain 2**		**Domain 3**		**Domain 4**	
	**Physical**		**Psychological**		**Social relationship**		**Environment**	
	**Mean ± SD**	**p value**	**Mean ± SD**	**p value**	**Mean ± SD**	**p value**	**Mean ± SD**	**p value**
No of positive urine tests*	1.02 ± 1.77	r = 0.213	1.02 ± 1.77	r = 0.115	1.02 ± 1.77	r = 0.066	1.02 ± 1.77	r = 0.130
		p = **0.019**		p = 0.206		p = 0.467		p = 0.153
Education level**								
Primary (9)	5.22 ± 15.41	df1 = 3	5.33 ± 20.94	df1 = 3	−3.44 ± 15.13	df1 = 3	3.33 ± 15.68	df1 = 3
Junior high (44)	14.00 ± 15.13	df2 = 118	18.64 ± 18.71	df2 = 118	7.64 ± 18.23	df2 = 118	8.66 ± 13.50	df2 = 118
Senior high (65)	13.42 ± 17.45	F = 1.622	13.32 ± 20.23	F = 3.403	5.77 ± 22.63	F = 7.785	10.15 ± 15.80	F = 1.718
Tertiary (4)	27.00 ± 23.58	p = 0.188	40.50 ± 33.97	p = **0.020**^**#**^	53.25 ± 35.95	p < **0.001**	23.50 ± 21.21	p = 0.167
HIV Status **								
Negative (95)	14.73 ± 17.68	df1 = 2	18.22 ± 20.78	df1 = 2	9.49 ± 22.96	df1 = 2	11.74 ± 15.38	df1 = 2
Positive (21)	10.10 ± 13.59	df2 = 119	7.81 ± 17.78	df2 = 119	1.67 ± 18.53	df2 = 119	2.76 ± 11.98	df2 = 119
Unknown (6)	5.33 ± 9.03	F = 1.398	0.17 ± 20.65	F = 4.072	−7.33 ± 26.78	F = 2.388	−1.33 ± 14.65	F = 4.847
		p = 0.251		p = **0.019**		p = 0.096		p = **0.009**

*Pearson correlation, **ANOVA test, significant when p value < 0.05.

^#^Post hoc performed using Scheffe method.

Stepwise multiple linear regression analysis was performed to identify variables that uniquely predicted each domain of quality of life. For the physical domain, there was no significant predictor found for the improvement of quality of life. For both the psychological and social domains, having tertiary education is a significant predictor for improvement in both aspects of quality of life (F = 6.027, p = 0.014; F = 19.496, p < 0.001 respectively). Positive HIV status is the only significant predictor (with negative correlation) for the environment domain (F = 5.166, p = 0.025). The model summaries for improvement in the three domains are as follows:

 Psychological = 14.70 + 25.81(Tertiary Education)

 Social relationship = 5.76 + 47.49(Tertiary Education)

 Environment = 10.96 – 8.20(HIV Positive)

## Discussion

The high prevalence of male Malay opioid abusers in MMT programme is similar to findings in other studies and reports in Malaysia [9,12,22,23]. It is a reflection of the national data, where the prevalence of Malay drug abusers up to December 2009 was 87.09% [24]. The majority clients (43.4%) were from the age group 30 – 39 years old when they first enrolled into MMT programme in the district of Tampin. However, a 2009 report from the National Anti-Drug Agency, Malaysia showed that the highest prevalence of drug abusers is among the age group of 20 – 29 years [24]. There are several possible reasons as to why there are far more mature opioid abusers seek drug substitution therapy. Perhaps, older clients have more awareness of their addiction problem and are keener to liberate themselves from this unstable and ill-fated lifestyle. They are also more likely to be in committed long term relationship, such as marriage and family. Having responsibilities on others would encourage them to seek stability in life through drug substitution therapy. Even for the younger age group in MMT programme, they more likely to have poor compliance towards treatment, in comparison to older age group [12]. This demonstrates the higher commitment shown by the older opioid abusers to find solutions for their addiction.

A recent review on quality of life among opiate-dependent individuals showed that this subpopulation had lower quality of life in comparison to the general population and the people with various medical illnesses [25]. Drug substitution therapy such as MMT, not only has its role in harm reduction, but it also has a beneficial impact on improving quality of life. This study has managed to show significant increasing trend of quality of life in all four domains measured; the physical, psychological, social relationships and environment, within 6 months of therapy. The findings are similar to other studies. Huong et al. showed similar trend for MMT clients who only had 3 to 6 months of therapy [22]. Padaiga et al. showed significant improvement at 6 months for physical, psychological, and environment domain but not for social relationships [26]. Recently, Xiao et al. demonstrated significant improvement of quality of life among MMT clients in China, as soon as 1 month of therapy [27]. Torrens et al. also showed marked increase in quality of life in the first year, followed by lesser but steady improvement for the next 3 years [28]. Our study has strengthened the conclusion that MMT has invaluable role in short term positive effect on clients’ quality of life. In his review, Maeyer et al. argued that the long term effect is still unclear [25] although we anticipate that the positive trend in improvement in quality of life will continue and plateau, perhaps as comparable as the general population. Further studies are needed to verify that the improvement of quality of life is sustained in the long run.

Our findings showed that the most improved quality of life domain was on psychological, followed by physical, environment and the least was social relationships. Huong et al. and Padaiga et al. also showed that social relationship has the least improvement among clients in MMT programme. In many parts of the world, opioid drug users are socially marginalised, so this might explain why it is harder for them to improve the social relationship quality in the short term of drug substitution therapy. Perhaps, the MMT programme should include more interventions on enhancing clients’ social and relationship skills. On a greater scale, the local community and the public should be encouraged to assimilate opioid dependent persons into the society.

Quality of life is subjective to the individual’s perceptions. Nevertheless, in this study, we attempted to identify possible factors that were associated with improvement of quality of life. We found that educational level has a significant effect on growth in psychological and social relationship domains. For these domains, clients with tertiary education have higher improvement after 6 months, in comparison to primary and secondary education. We do not find any other study with similar findings. Possible explanation may include that persons with higher educational level have better coping strategies and have higher earnings, thus contributing to better quality of life. We suggest that future research to include the level of income to determine whether this has a significant effect on improvement of quality of life. An objective measurement of clients’ quality of life through in depth interviews and home visits may provide a more realistic view on the impact of MMT programme on the clients’ quality of life.

In this study, we found that HIV status was associated with the changes in quality of life for the psychological and environment domain. This finding is in accordance to a study done by Fernandez-Miranda et al. in which they found that HIV positive MMT clients have lower quality of life scores [29]. HIV and AIDS are associated with poorer health status and depression thus maybe reflected in the quality of life assessment. A recent finding by Lee et al. showed that HIV status did not significantly affect the quality of life of MMT clients [30].

The implication on these findings may be that MMT programme especially the non-pharmacological interventions, such as psychosocial programmes, are able to focus on certain subgroups (e.g. from lower educational level and those who are HIV positive) in order to further improve their quality of life.

The previous detoxification programmes for opioid abusers in Malaysia had yielded little success. Now, the Malaysian Ministry of Health is improving its commitment in battling substance abuse problem in Malaysia by increasing the number of dedicated MMT clinics in primary health care. This study has provided some basis of support for this decision.

This study provides a small but important evidence of the effectiveness of MMT programme in Malaysia. Even though the programme is fairly new, there is already an early indication to support its continuity and expansion. Nonetheless, this study is limited by having only one assessment post MMT intervention, i.e*.* at 6 month as well as small number of clients. It would be nice to cohort and identify the trend of quality of life in these clients for a longer period of time. This study also only examine the scoring of quality of life assessment, while it may be useful to have additional qualitative evaluation such as in depth interview with the clients.

## Conclusion

MMT clients who completed 6 months of therapy showed significant improvement in the quality of life in the district of Tampin, Negeri Sembilan, Malaysia. In implementing the non-pharmacotherapy interventions on the clients, additional focus should be given to certain groups of MMT clients to ensure the fullest potential in improving their quality of life. Interventions could also be aimed at strengthening clients’ social and relationship skills. The MMT programme has great prospects in the treatment of opioid addiction, of which is still a big public health predicament in Malaysia.

## Abbreviations

MMT: Methadone maintenance therapy;HIV: Human immunodeficiency virus;WHOQOL-BRE:, WHO quality of life questionnaire (Abbreviated version);OTI: Opiate treatment index.

## Competing interests

The authors declare that they have no competing interests.

## Authors’ contributions

NB was involved in the research design, carried out the data collection, and drafted the manuscript. SA and MRH were involved in statistical analysis and drafted the manuscript. NA was involved in clinical arrangement, the data collection and drafted the manuscript. All authors read and approved the final manuscript.

## References

[B1] WHO Proposal for the inclusion of Methadone in the WHO model list of essential medicines 2004 Geneva: World Health Organization http://www.who.int/substance_abuse/activities/methadone_essential_medicines.pdf

[B2] Walwyn WM Miotto KA Evans CJ Opioid pharmaceuticals and addiction: The issues, and research directions seeking solutions Drug and Alcohol Dependence 2010 108 156 165 10.1016/j.drugalcdep.2010.01.001 20188495 PMC3072810

[B3] Ward J Hall W Mattick RP Role of maintenance treatment in opioid dependence Lancet 1999 353 221 226 10.1016/S0140-6736(98)05356-2 9923893

[B4] Lee S The contribution of methadone maintenance treatment to HIV prevention – the case of Hong Kong International Conference on Tackling Drug Abuse - Conference Proceedings. Hong Kong: Narcotics Division 2005 191 205 http://www.nd.gov.hk/en/conference_proceedings/Drugs_proBK_Part3/Drugs_proBK_LeeSS.pdf. Accessed 12/01/2010

[B5] Weber R Ledergerber B Opravil M Siegenthaler W Lüthy R Progression of HIV infection in misusers of injected drugs who stop injecting or follow a programme of maintenance treatment with methadone BMJ 1990 301 1362 1365 10.1136/bmj.301.6765.1362 1980219 PMC1664524

[B6] Sorensen JL Copeland AL Drug abuse treatment as an HIV prevention strategy: a review Drug and Alcohol Dependence 2000 59 17 31 10.1016/S0376-8716(99)00104-0 10706972

[B7] Nicholls L Bragaw L Ruetsch C Opioid dependence treatment and guidelines J Manag Care Pharm 2010 16 1-b S14 S21 20146550 10.18553/jmcp.2010.16.S1-B.14 PMC10437806

[B8] Farrell M Ward J Mattick R Hall W Stimson GV des Jarlais D Gossop M Strang J Fortnightly review: methadone maintenance treatment in opiate dependence: a review BMJ 1994 309 997 1001 10.1136/bmj.309.6960.997 7950725 PMC2541312

[B9] Rusdi AR Noor Zurani MHR Muhammad MAZ Mohamad HH A fifty-year challenge in managing drug addiction in Malaysia Journal of University of Malaya Medical Centre 2008 11 3 6

[B10] Ministry of Health Malaysia National methadone maintenance therapy guideline 2005 1 Malaysia: MOH

[B11] Liow TL The Malaysia national conference on HIV/AIDS in conjunction with world AIDS day 2009 (speech text) http://blogmoh.moh.gov.my/the-malaysia-national-conference-on-hivaids-in-conjunction-with-world-aids-day-2009/. Accessed 05/02/2010

[B12] Sharifa Ezat W Noor Azimah H Rushidi R Raminder K Ruhani I Compliance towards methadone maintenance therapy and its associated factors in Selangor primary care centers and Kuala Lumpur hospital Med J Malaysia 2009 64 65 70 19852326

[B13] Guggenmoos-Holzmann I, Bloomfield K, Brenner H, Flick U Quality of life and health: concepts, methods and applications 1995 Oxford: Blackwell Science

[B14] Fitzpatrick R Fletcher A Gore S Jones D Spiegelhalter D Cox D Quality of life measures in health care. I: Applications and issues in assessment BMJ 1992 305 1074 1077 10.1136/bmj.305.6861.1074 1467690 PMC1883623

[B15] Carr AJ Gibson B Robinson PG Measuring quality of life: Is quality of life determined by expectations or experience? BMJ 2001 322 1240 1243 10.1136/bmj.322.7296.1240 11358783 PMC1120338

[B16] Ministry of Health Malaysia: National policy and standard operating procedures for Methadone Maintenance Therapy 2005 1 Malaysia: MOH

[B17] WHO WHOQOL Measuring quality of life Geneva: World Health Organization http://www.who.int/mental_health/media/68.pdf. 1997. Accessed 13/01/2010

[B18] WHO WHOQOL-BREF Introduction, administration, scoring and generic version of the assessment Geneva: World Health Organization http://www.who.int/mental_health/media/en/76.pdf. 1996. Accessed 13/01/2010

[B19] Hasanah CI Naing L Rahman AR World Health Organization Quality of Life Assessment: brief version in Bahasa Malaysia Med J Malaysia 2003 58 79 88 14556329

[B20] Darke S Ward J Hall W Heather N Wodak A The opiate treatment index (OTI) manual 1991 Australia: National Drug and Alcohol Research Centre http://www.med.unsw.edu.au/NDARCWeb.nsf/resources/TR_29/$file/TR.011.pdf. Accessed 13/01/2010

[B21] WHO WHOQOL User manual 1998 Geneva: World Health Organization http://www.who.int/mental_health/media/en/620.pdf

[B22] Huong AGW Ng CG Amer SA Quality of life assessment of opioid substance abusers on methadone maintenance therapy (MMT) in University Malaya Medical Centre ASEAN Journal of Psychiatry 2009 10 1 11

[B23] Ramli M Nora MZ Zafri AA Junid MR Umeed AK Hajee MI High risk behaviours and concomitant medical illness among patients at Methadone Maintenance Therapy clinic, Hospital Tengky Ampuan Afzan, Malaysia Malaysian Family Physician 2009 4 2&3 77 82 http://www.e-mfp.org/2009v4n2_3/Methadone_Maintenance_Theraphy_Clinic.html Accessed 18/04/2010 PMC4267043 25606168

[B24] National Anti-Drug Agency Drug Report December 2009 (Malay language) http://www.adk.gov.my/html/laporandadah/laporandadahdis09.pdf National Anti-Drug Agency, Malaysia. Accessed 18/04/2010

[B25] De Maeyer J Vanderplasschen W Broekaert E Quality of life among opiate-dependent individuals: A review of the literature International Journal of Drug Policy 2010 21 364 380 10.1016/j.drugpo.2010.01.010 20172706

[B26] Padaiga Z Subata E Vanagas G Outpatient methadone maintenance treatment program. Quality of life and health of opioid-dependent persons in Lithuania Medicina (Kaunas) 2007 43 235 241 17413253

[B27] Xiao L Wu z Luo W Wei X Quality of life of outpatients in Methadone Maintenance Treatment clinics J Acquir Immune Defic Syndr 2010 53 S116 S120 20104102 10.1097/QAI.0b013e3181c7dfb5 PMC2818825

[B28] Torrens M Domingo-Salvany A Alonso J Castillo C San L Methadone and quality of life Lancet 1999 353 1101 10199379 10.1016/S0140-6736(05)76462-X

[B29] Femandez-Miranda JJ Gonzalez MP Saiz PA Gutierrez E Bobes J Quality of life, psychopathological status and drug use in a methadone maintenance treatment in Spain Eur Neuropsychopharmacol 1998 8 S285

[B30] Lee TSH Shen HC Wu WH Huang CW Yen MY Wang BE Chuang P Shih CY Chou YC Liu YL Clinical characteristics and risk behavior as a function of HIV status among heroin users enrolled in methadone treatment in northern Taiwan Substance Abuse Treatment, Prevention, and Policy 2011 6 6 10.1186/1747-597X-6-6 PMC3079677 21473789

